# Supplementary material for: Impaired NRF2 Inhibits Recovery from Ischemic Reperfusion Injury in the Aging Kidney
Source: Antioxidants (Basel). 2023 Jul 18;12(7):1440. doi: 10.3390/antiox12071440 (PMC10376352; doi:10.3390/antiox12071440)
Supplement: Supplementary file 1 [file antioxidants-12-01440-s001.zip › antioxidants-2486574-supplementary.pdf]

Supplementary Materials

Impaired NRF2 Inhibits Recovery from Ischemic Reperfusion Injury in the Aging Kidney

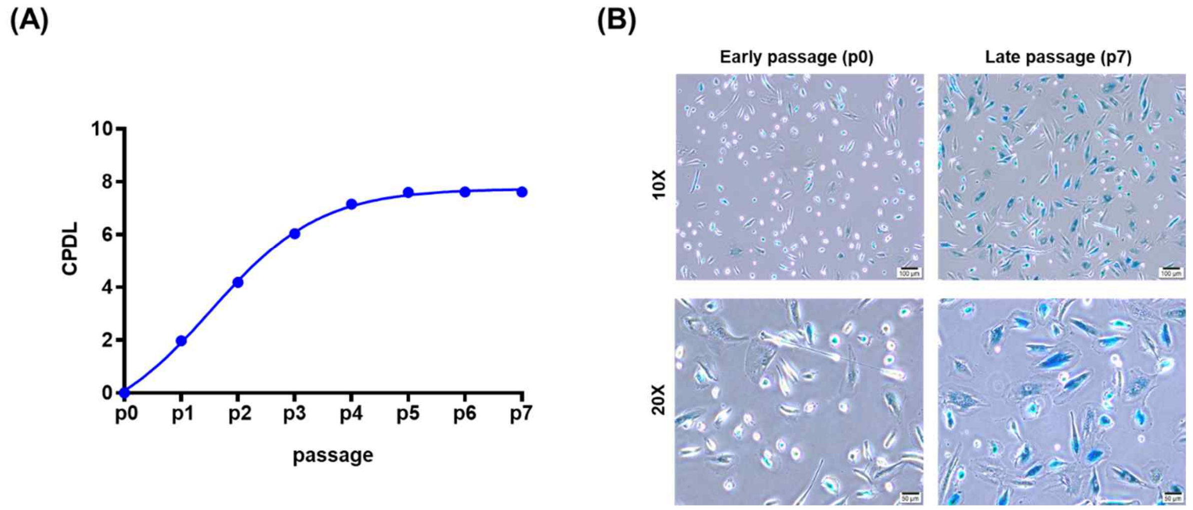

Figure S1. Establishment of senescent RPTECs. (**A**) Growth curve of senescent RPTECs. CPDL was measured from passages 0 to 7. (**B**) Representative images of passage 1 and passage 7 RPTECs. SA-β-gal staining was performed using a Senescence β-Galactosidase Staining Kit. The senescent cells are blue. Abbreviations: RPTEC, primary renal proximal tubule epithelial cell; CPDL, cumulative population doubling level; SA-β-gal, senescence-associated beta-galactosidase.

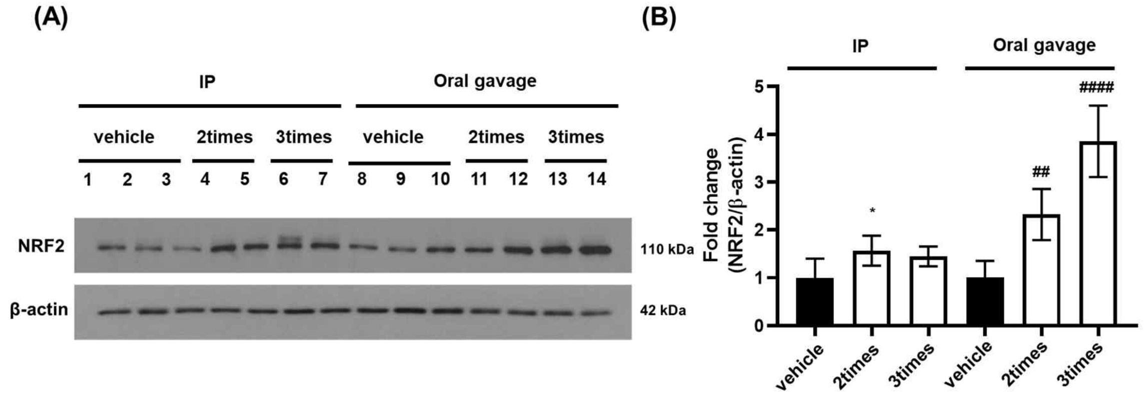

Figure S2. Comparison of intraperitoneal injection and oral gavage for CDDO-Me treatment. Kidney tissues from mice were homogenized and NRF2 expression in the tissue lysate was estimated by western blot analysis. β-actin was used as the control. Representative blots are shown on the left (**A**), and the densitometry of the blots is

shown on the right (**B**). Error bars represent standard deviations. *p < 0.05 vs. IP vehicle treated group, ##p < 0.01, ####p < 0.0001 vs. oral gavage vehicle treated group. Abbreviations: CDDO-Me, bardoxolone methyl; IP, intraperitoneal injection group; 2 times, 2 times a week; 3 times, 3 times a week.

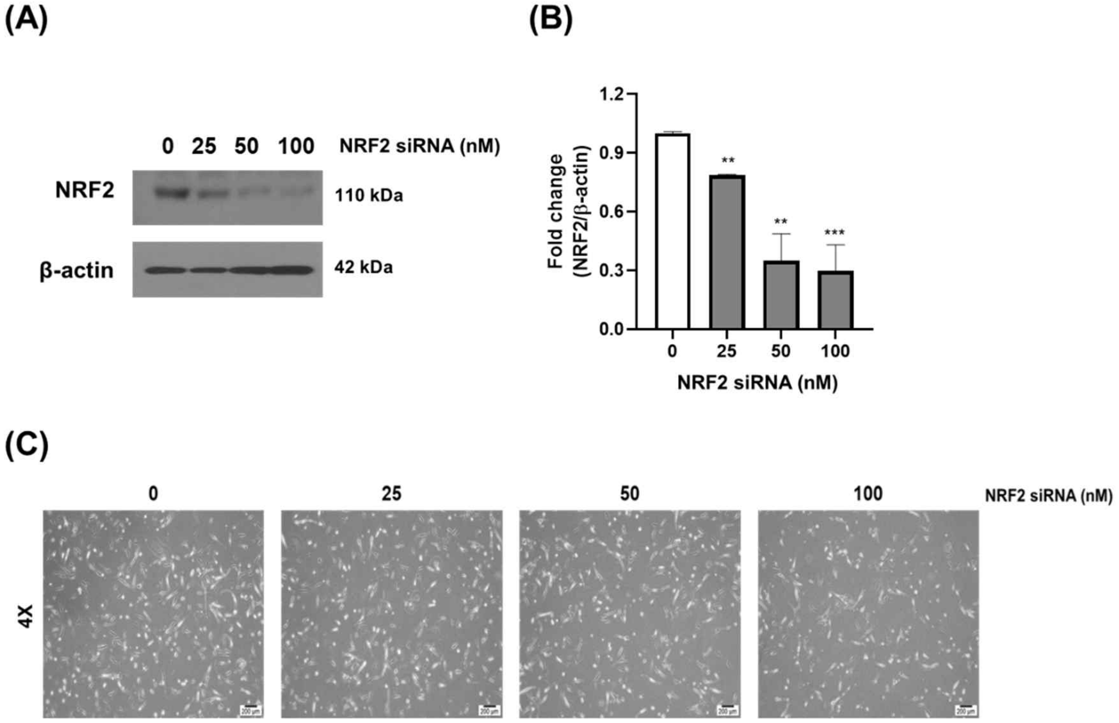

Figure S3. Inhibiting NRF2 in RPTECs with NRF2 siRNA. RPTECs were transfected with 0, 25, 50, and 100 nM of NRF2 siRNA for 24 h. NRF2 expression was evaluated by western blot analysis. β-actin was used as the control. Representative blots are shown on the left (**A**), and the densitometry of the blots is shown on the right (**B**). Error bars represent standard deviations. **p < 0.05, ***p<0.01 vs. 0 nM. (**C**) Representative images of transfected RPTECs. Abbreviations: NRF2, nuclear factor erythroid-2-related factor; siRNA, small interfering RNA; RPTEC, primary renal proximal tubule epithelial cell.
